# A rare case of a mammary analog secretory carcinoma of the right submandibular gland

**DOI:** 10.1093/jscr/rjaf103

**Published:** 2025-02-28

**Authors:** Maria R Bianco, Cosimo Galletti, Carlo Gentile, Olindo Di Benedetto, Sofia Pepe, Anna M Lavecchia, Eugenia Allegra

**Affiliations:** Otolaryngology, Department of Health Science, University of Catanzaro, 88100 Catanzaro, Italy; Otolaryngology, Faculty of Medicine and Surgery, Kore University of Enna, Enna, Italy; Pathological Anatomy Unit, “Renato Dulbecco” Hospital, 88100 Catanzaro, Italy; Neuroradiology Unit, University of Catanzaro, 88100 Catanzaro, Italy; Otolaryngology, Department of Health Science, University of Catanzaro, 88100 Catanzaro, Italy; Pathological Anatomy Unit, “Renato Dulbecco” Hospital, 88100 Catanzaro, Italy; Otolaryngology, Department of Health Science, University of Catanzaro, 88100 Catanzaro, Italy

**Keywords:** MASC, salivary gland tumor, submandibular gland, head & neck, cancer

## Abstract

Salivary gland cancer is one of the most common malignant neoplasms of head and neck. Not only is it rare but its clinical course is also very heterogeneous. A 64-year-old woman was diagnosed with submandibular gland carcinoma. We performed an ultrasound examination and a magnetic resonance imaging of the head and neck, which revealed an oval expansive process in the right submandibular salivary gland, indicative of an aggressive neoplasm, such as adenocarcinoma. Surgical excision and histological examination were conducted reporting macroscopically a whitish nodular formation measuring 1.5 × 1 cm and microscopically positivity to cytokeratin 7 and mammaglobin, compatible with a mammary analog secretory carcinoma. No N positive since there is no clinical, instrumental, or laboratory evidence of the primary pathology. We just performed a surgical excision and accurate follow-up visits, and the patient has been free from disease for 2 years. Major efforts should be spent to establish the most adequate management and follow-up protocol visits.

## Introduction

Mammary analog secretory carcinoma (MASC) of the salivary glands is a low-grade tumor of the salivary glands of the head and neck region that has been recently reported and accounts for 4%–4.5% of all tumors. Malignancies of the salivary glands [1, 2].

MASC is an interesting and rare malignant tumor of the salivary glands. It was first described in 2010 and added to the Milan Salivary Gland Cytopathology Reporting System (MSRSGC). This system was published in 2018 and has been increasingly recognized by various pathologists and institutions until it has become a universal language for reporting salivary gland cytology [3, 4]. Secretory breast cancer (SBC) shares some characteristics, such as histology, immunohistochemical, and genetic patterns [5, 6].

MASCs are most commonly found in the parotid gland but sometimes interest lips, soft palate, and submandibular salivary glands [7]. They usually present as painless nodules that grow slowly but progressively [6, 8].

Although cytopathological diagnosis is challenging, classification is important in determining the choice of surgical treatment, and most MASCs are considered low-grade malignant, approached with complete *exeresis* and a good long-term prognosis. As described in the scientific literature, the 5-year overall survival rate for patients with MASC is 95%, the 5-year disease-free survival rate is 89%, and the incidence of lymph node metastasis is low. However, few high-grade lymph node or distant metastasis cases have been reported [2].

The diagnosis of MASC and its differential diagnosis from other tumors is based on the operative sample examination, performing immunohistochemistry, and genetic tests, showing positivity for mammaglobin, S-100 protein, and PAS-diastase but negative for DOG-1. Immunohistochemical tests, preferably supplemented by molecular biology tests, are required to confirm the diagnosis; immunohistochemical similarities between MASC and SBC include S100 protein +, epithelial membrane antigen (EMA), and vimentin — MASC (ER/PR/Her---) has been reputed as a low-grade tumor showing microcystic, papillary cystic, ductal and glandular enhancing features. The cells have pale nuclei, granular or vacuolated eosinophilic cytoplasm, and show intraductal or intracytoplasmic secretion [6, 9, 10].

MASC is a specific tumor characterized by a precise genetic mutation, a rearrangement of the ETV6 gene; MASC is the result of a recurrent balanced chromosomal translocation t(12;15)(p13;q25) resulting in a favorable gene fusion of the ETV6 gene on chromosome 12 and the NTRK3 gene on chromosome 15. This fusion likely gives rise to an active chimeric tyrosine kinase that activates the mitogenic RAS-MAP kinase pathway and the phosphatidylinositol-3-kinase-AKT pathway. Genetic alterations are observed by ETV6 fluorescence in situ hybridization (FISH) or detection of ETV6-NTRK3 fusion transcripts by reverse transcription polymerase chain reaction (RT-PCR) [5, 11].

The MASC physiopathology attitude varies from slow-growing tumors that rarely reoccur after surgical resection to highly invasive forms that lead to systemic metastases. In many surgical histological samples, MASC is erroneously classified as Acinic Cell carcinoma (AciCC), mucoepidermoid carcinoma (MEC), and unspecified adenocarcinoma [3, 11, 12].

## Case report/case presentation

The following case concerns a 64-year-old woman with a MASC of the submandibular gland.

The patient presented an increase in the volume of the gland, which appeared hard and tense on palpation but not painful. An ultrasound revealed in the right submandibular area an oval formation with clear and lobulated contours, slightly hypoechoic with some areas of colliquation, compatible, in the first hypothesis with the diagnosis of pleomorphic adenoma and, in the second hypothesis, with a cystadenolymphoma.

**Figure 1:**
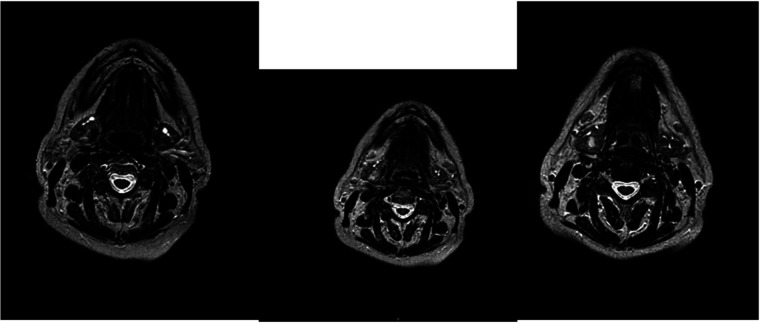
MRI with the contrast medium which highlighted the presence of an ovoid expansive process of ⁓15 mm in diameter with undefined margins compared to the surrounding glandular parenchyma. The lesion was characterized as an area with a non-homogeneous signal for T2 iso-hyperintensity and T1 iso-hypointensity with equally heterogeneous enhancement after administration of the contrast medium.

This hypothesis was questioned by magnetic resonance imaging (MRI), which highlighted the presence of an ovoid expansive process of ⁓15 mm in diameter with undefined margins compared to the surrounding glandular parenchyma. The MRI characteristics suggested an aggressive lesion such as adenocarcinoma: the lesion was characterized as an area with a non-homogeneous signal for T2 iso-hyperintensity and T1 iso-hypointensity with equally heterogeneous enhancement after administration of the contrast medium (Fig. 1).

**Figure 2:**
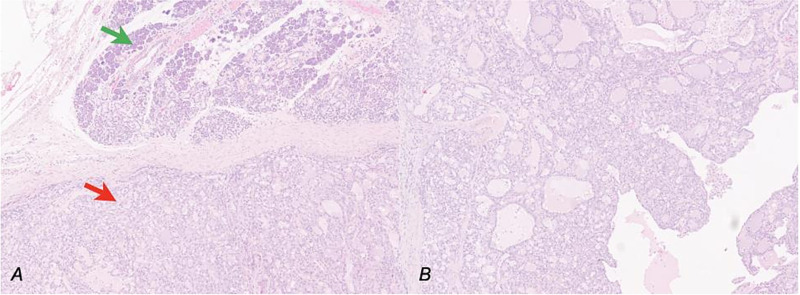
Secretory carcinoma of the salivary gland H&E stained section (10HPF). (a) Transition point between normal salivary gland tissue (upper arrow) and neoplastic tissue (lower arrow). (b) Some architectural details of neoplasia: Solid, glandular, and cystic areas and papillary extroflections.

**Figure 3:**
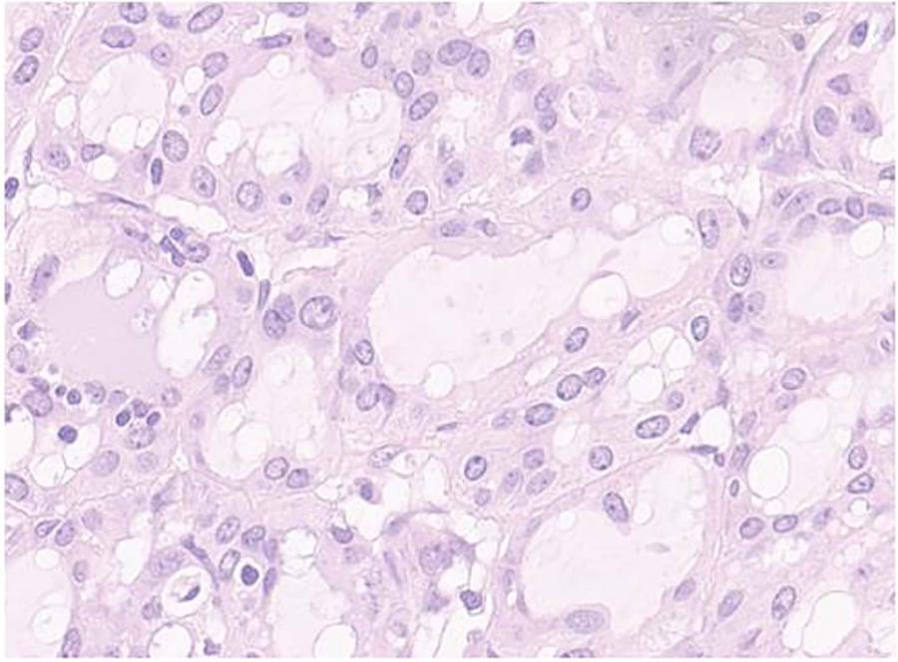
Secretory carcinoma of the salivary gland H&E stained section (40HPF). Tumors aggregate with solid and glandular structure compound of neoplastic cell with eosinophilic vacuolated cytoplasm and monomorfic round vescicular nucley with small nucleoly.

**Figure 4:**
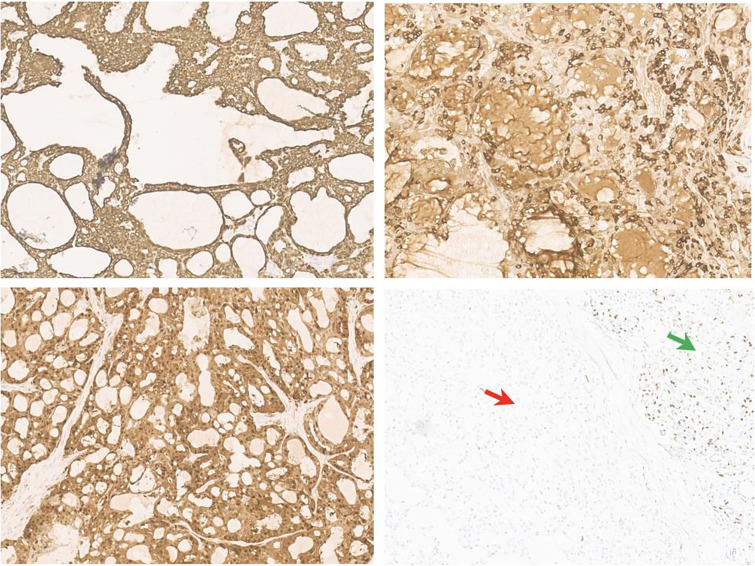
Antibodies inmunohistochemical neoplasia panel (10HPF). (a) Intense membrane cycheratin 7 staining of the neoplastic cell. (b) Citoplasmatic and membranes mammoglobin staining of the neoplastic cell. (c) Diffuse citoplasmatic and nuclear S100 staining of the neoplastic cell. (d) The possibility of the p63 antibody staining in the normal salivary gland tissue (right arrow) and negativity of neoplastic population (left arrow).

Therefore, surgical excision and histological examination were conducted. The latter reported macroscopically a whitish nodular formation measuring 1.5 × 1 cm and positivity to cytokeratin 7, S100, and mammaglobin, compatible with a MASC, and surgical margins free from invasion (Figs 2–4).

The patient underwent every 3 months an ultrasound for the first 12 months, as well as a CT neck and chest at the 6th and 12th month after the surgery. In the 2nd year of follow-up, the patient underwent a visit every 3 months, an ultrasound every 6 months, and an annual CT scan.

The patient presented a favorable outcome with free-from disease with a 24-month follow-up period.

## Discussion

In 2010, Skalova *et al.* described the first salivary gland secretory carcinoma in a series of 16 salivary gland tumors [13]. Two main hypotheses could justify the presence of mammary tissue in the submandibular gland: the presence of metastasis from breast secretory carcinoma or embryonic remnants from an ectodermal transition defect. The former hypothesis is unlikely to be supported due to the lack of clinical, instrumental, or laboratory evidence of primary pathology. However, the scientific literature evidenced the presence of PAX-8 as a marker of distant metastasis of breast cancer, including secretory histologic types. The presence of PAX-8 is not known as a marker of distant metastasis of breast cancer [14]. In contrast, the hypothesis of embryologic remnants seems more likely since mammary and salivary glands share the same embryologic origin and both derive from the ectoderm [9]. Salivary gland carcinomas share the same histological and immunohistochemical features as mammary gland secretory carcinomas, including mutated ETS 6 (ETV6) gene and translocation of neurotrophic receptor tyrosine kinase type 3 (NTRK3), which regulate cell growth and differentiation. This genetic alteration is peculiar for MASC in the context of salivary gland tumor [3, 9, 14, 15]. MASC cells are typically lobular, with macro- and microcysts and, less frequently, solid and papillary. The cytoplasm is vacuolar or eosinophilic, and PAS/D-positive intracytoplasmic “glassy” excretions are present in the cyst lumen. The tumor cells also express CK7, CK8, CK18, CK19, GCDFP15, and EMA, but not specific [16]. The immunological profile suggests a myotubular origin of MASC; IHC staining is a very specific method to differentiate MASC from other salivary gland tumors; the diagnosis of MASC is made in the presence of classic histological forms with double positive immunostaining for mamaglobin and S-100 [3, 10]. Frequently, histopathological and immunohistochemical studies do not guarantee a definitive diagnosis of MASC [2, 9]. The most affected site is the parotid salivary gland for MASC, but it can also affect the minor salivary glands, and minor salivary glands of lips, soft palate, and neck [7, 11, 12]. Regarding biological behavior, MASC has lymphofilia of regional lymph node metastases than salivary adenocellular carcinoma (AciCC). Gender differences are not available in literature [14, 16]. Various imaging modalities such as cervical ultrasonography and contrast-enhanced cervical CT/MRI are also helpful in the diagnosis. These imaging modalities help in the preoperative evaluation of tumor size, vascular and lymph node metastasis, and proximity to major blood vessels. New diagnostic tools such as immunohistochemical tests for mammaglobin, vimentin, and S-100, in combination with fine needle aspiration testing, allow a definitive diagnosis. Hormonal tests, especially those based on ER, PR, and HER-2 receptor status, are used in selective studies [1, 4, 16]. FDG-PET/CT is not indicated in head and neck cancer patients due to a lack of clear guidelines and limited accessibility due to high costs and availability.

There is no clear indication supporting the adjuvant chemotherapy after surgical resection.

A high number of patients were treated based on histochemical similarities and growth patterns with other low-grade malignancies and AciCC and underwent surgery with or without radiotherapy or postoperative adjuvant chemoradiotherapy. Primary radiotherapy or chemoradiotherapy was not considered a common treatment option, as evidenced by the literature. According to literature, a clinical safety margin of 10 mm is considered sufficient for adequate tumor resection and is mandatory for squamous cell carcinoma of head and neck [8, 17].

There are no confirmed data on whether selective neck dissection improves outcome and overall survival in patients with negative lymph nodes [3, 6]. The likelihood of metastasis or recurrence after surgical resection is strictly related to the tumor invasion at the time of presentation. Since perineural and lymphatic invasion is rare, the prognosis remains favorable according to the current medical literature [18, 19]. Level I, II, III, and Va, like other malignant tumors of the oral cavity, are prone to lymph node metastasis from MASCs. In case of positive lymph node metastasis, levels I-V require unilateral or bilateral neck dissection and adjuvant radiotherapy or chemoradiotherapy due to the invasive nature of the tumor. In low-grade tumors without lymph node metastases, surgery alone may be sufficient [1, 3, 20]. Elective TRKI therapy may be offered for the treatment of patients with locally advanced, metastatic, or no susceptible to surgical treatment. The most proven and approved agents are larotrectinib and entrectinib. TRKIs such as citravatinib, cabozantinib, and belizatinib have also been studied in clinical cases [1, 2]. Disease-free periods registered in literature for MASC range from 71 to 115 months, more or less than the 92 to 148 months reported for AciCC [6, 16]. Differential diagnoses include low-grade MEC defined by the presence of various cell types in varying proportions, including squamous cells, clear cells, mucous cells, carcinoma cells, intermediate cells, columnar cells, and may also present with sclerotic fibrous stroma and mucinous effusion [9, 21]. It is recommended that the first imaging scan should be performed at 6 months postoperatively and annually until the end of follow-up. During the first 2 years and in high-risk patients, as in our case, the time gap between evaluations should be rigid: one every 3–4 months [2, 22, 23].

## Conclusion

Although there are no guidelines for the treatment of MASC, literature reports that surgery should be performed only if it is necessary to treat local tumors without lymph nodes or systemic metastases. However, in case of advanced tumor behavior or lymph node metastases, elective neck dissection should be considered even if lymph node metastases are negative. If lymph node metastases are present, ipsi or bilateral I-V neck dissection and adjuvant radiotherapy or chemoradiotherapy may be necessary. The benefit of chemotherapy remains unclear. Novel monoclonal antibody therapies with TRKIs, such as ralotrectinib and entrectinib, may represent therapeutic for inoperable cases, emphasizing the need of molecular diagnostics in particular situations of a high variety of cellular lines. Standardized guidelines on the role of surgical treatment, radiotherapy and chemotherapy, timing and follow-up methods, regional lymph node metastases, distant metastases, recurrence, and MASC-related mortality are also needed.

## Data Availability

The data that support the findings of this study are available from the corresponding author [CG], upon reasonable request.
